# Myelitis and Pulmonary Tuberculosis

**Published:** 1905-08-19

**Authors:** 


### MYELITIS AND PULMONARY TUBERCULOSIS.

CLÉMENT[1] records a series of cases of pulmonary tubercle with which were associated evidences of disease of the nerve roots and spinal cord, and suggests that the nervous lesions were due to the tuberculous toxins. In one instance the patient exhibited the symptoms and signs of disseminated sclerosis, together with advanced disease in the larynx and lungs. The other cases, five in number, were examples of tabes dorsalis, as shown by the absence of knee-jerks, and by Romberg's sign and the Argyll-Robertson pupil. An interesting feature of this group was the marked tendency of the nervous disease to remain stationary, the lightning pains being severe, but not accompanied by the development of ataxia. In one of the cases observation of these facts had been continued over a period of 20 years, the lung trouble in this instance having subsided. Among the other cases were two examples of rapid extension leading to a fatal issue from phthisis pulmonalis, but for the most part the pulmonary tubercle was of the latent or chronic order. Dr. Ernst Oberndörfer[2] has reported a case which may be associated with the above records. The patient was a man of 26 years, the victim of pulmonary tuberculosis. Some considerable time after this diagnosis had been made he began to complain of difficulty of micturition and defæcation, with pains in the epigastric and lumbar regions. Later there were paresis of the left lower limb and partial of anæsthesia over the lower half of the body, and ultimately retention of urine and complete paraplegia. A diagnosis of tuberculosis of the spinal cord was made, in opposition to the suggestion of compression paraplegia, on the following grounds:—The existence of tubercular disease of the lungs; the absence of objective evidence of vertebral caries; and the manner of commencement and spread of the cord symptoms—at first unilateral paralysis and patches of anæsthesia with, later, bilateral motor and sensory defect. The autopsy revealed a normal condition of the vertebræ and meninges and a caseous mass containing giant cells and tubercle bacilli in the substance of the spinal cord. This tumour occupied the whole of the left side and extended into the right side of the cord at the level of the eighth thoracic segment; it also extended downwards in a conical form and ended at the ninth thoracic segment. Only a few parallel records are to be found in medical literature.

[1] Lyon Med., quoted in Brit. Med. Jour. Epit., July 22, 1905.

[2] Münchener Medicinische Wochenschrift, quoted in Lancet, April 16, 1904.

